# Beneficial effects of physical exercise and an orally active mGluR2/3 antagonist pro-drug on neurogenesis and behavior in an Alzheimer's amyloidosis model

**DOI:** 10.3389/frdem.2023.1198006

**Published:** 2023-09-06

**Authors:** Georgina Perez Garcia, Mesude Bicak, Jacqueline Buros, Jean-Vianney Haure-Mirande, Gissel M. Perez, Alena Otero-Pagan, Miguel A. Gama Sosa, Rita De Gasperi, Mary Sano, Fred H. Gage, Carrolee Barlow, Joel T. Dudley, Benjamin S. Glicksberg, Yanzhuang Wang, Benjamin Readhead, Michelle E. Ehrlich, Gregory A. Elder, Sam Gandy

**Affiliations:** ^1^Department of Neurology, Icahn School of Medicine at Mount Sinai, New York, NY, United States; ^2^Research and Development, James J. Peters Veterans Affairs Medical Center, Bronx, NY, United States; ^3^Department of Genetics and Genomic Sciences, Icahn School of Medicine at Mount Sinai, New York, NY, United States; ^4^Hasso Plattner Institute for Digital Health at Mount Sinai, Icahn School of Medicine at Mount Sinai, New York, NY, United States; ^5^Department of Psychiatry and Alzheimer's Disease Research Center, Icahn School of Medicine at Mount Sinai, New York, NY, United States; ^6^Laboratory of Genetics, The Salk Institute for Biological Studies, La Jolla, CA, United States; ^7^BrainCells, Inc., La Jolla, CA, United States; ^8^E-Scape Bio, South San Francisco, CA, United States; ^9^Department of Developmental and Cell Biology, University of Michigan, Ann Arbor, MI, United States; ^10^Arizona State University-Banner Neurodegenerative Disease Research Center, Arizona State University, Tempe, AZ, United States; ^11^Department of Pediatrics, Icahn School of Medicine at Mount Sinai, New York, NY, United States; ^12^Neurology Service, James J. Peters Department of Veterans Affairs Medical Center, Bronx, NY, United States; ^13^Mount Sinai Center for Cognitive Health, Icahn School of Medicine at Mount Sinai, New York, NY, United States

**Keywords:** APP/PS1 mice, physical exercise, mGlu2/3 antagonist, neurogenesis, BDNF, AD amyloid pathology

## Abstract

**Background:**

Modulation of physical activity represents an important intervention that may delay, slow, or prevent mild cognitive impairment (MCI) or dementia due to Alzheimer's disease (AD). One mechanism proposed to underlie the beneficial effect of physical exercise (PE) involves the apparent stimulation of adult hippocampal neurogenesis (AHN). BCI-838 is a pro-drug whose active metabolite BCI-632 is a negative allosteric modulator at group II metabotropic glutamate receptors (mGluR2/3). We previously demonstrated that administration of BCI-838 to a mouse model of brain accumulation of oligomeric Aβ^E22Q^ (*APP*^*E*693*Q*^ = “*Dutch APP*”) reduced learning behavior impairment and anxiety, both of which are associated with the phenotype of *Dutch APP* mice.

**Methods:**

3-month-old mice were administered BCI-838 and/or physical exercise for 1 month and then tested in novel object recognition, neurogenesis, and RNAseq.

**Results:**

Here we show that (i) administration of BCI-838 and a combination of BCI-838 and PE enhanced AHN in a 4-month old mouse model of AD amyloid pathology (*APP*^*KM*670/671*NL*^*/PSEN1*^Δ*exon*9^= APP/PS1), (ii) administration of BCI-838 alone or with PE led to stimulation of AHN and improvement in recognition memory, (iii) the hippocampal dentate gyrus transcriptome of APP/PS1 mice following BCI-838 treatment showed up-regulation of brain-derived neurotrophic factor (BDNF), PIK3C2A of the PI3K-mTOR pathway, and metabotropic glutamate receptors, and down-regulation of EIF5A involved in modulation of mTOR activity by ketamine, and (iv) validation by qPCR of an association between increased BDNF levels and BCI-838 treatment.

**Conclusion:**

Our study points to BCI-838 as a safe and orally active compound capable of mimicking the beneficial effect of PE on AHN and recognition memory in a mouse model of AD amyloid pathology.

## Introduction

Physical exercise (PE) has important neuroprotective and pro-cognitive benefits (Buchman et al., 2019; Mortimer and Stern, 2019). One long-suggested mechanism underlying the beneficial effects of PE on central nervous system (CNS) function is its effect on neurogenesis in the adult hippocampal dentate gyrus (DG) and the subventricular zone (SVZ) of the lateral ventricles (Obernier and Alvarez-Buylla, 2019; Toda et al., 2019). In these regions, new neurons are generated and incorporated into existing neuronal circuits where they promote structural and functional plasticity. Stimulation of adult hippocampal neurogenesis (AHN) has been proposed as a central mechanism of adult brain plasticity and a potential therapeutic target in a variety of psychiatric and neurodegenerative conditions (Toda et al., 2019). Drugs such as antidepressants that affect monoaminergic systems consistently increase AHN (Hanson et al., 2011). The DG is critical for learning and memory, and altered AHN has been implicated in the pathogenesis of neurodegenerative diseases such as Alzheimer's disease (AD) (Lazarov and Marr, 2010; Llorens-Martin, 2018). A recent review of AHN in neurological diseases (Gage, 2021) highlighted a key study of the DG from postmortem samples of patients who died from neurodegenerative disorders. The study demonstrated that functions of the neurogenic niche shifted and the cells produced were abnormal in shape and differentiation, further emphasizing the plasticity characteristic of the hippocampus and its potential as a therapeutic target (Terreros-Roncal et al., 2021). Furthermore, a recent study demonstrated that greater physical activity was associated with higher levels of presynaptic proteins, suggesting maintenance or building brain resilience (Casaletto et al., 2022).

Exercise-induced AHN has been extensively studied in rodents, and voluntary PE (e.g., running wheels) has been shown to enhance AHN in the DG (Ma et al., 2017). Studies utilizing cell fate tracers [e.g., bromodeoxyuridine (BrdU)], have shown that exercise increases proliferation of progenitor cells in the subgranular zone and promotes their survival and differentiation into mature neurons (Van Praag et al., 1999; Buchman et al., 2019). Increased expression of brain-derived neurotrophic factor (BDNF) may be one mechanism underlying the proneurogenic effect of PE (Liu and Nusslock, 2018).

Altered AHN has been reported in postmortem brain from humans suffering from AD (Lazarov and Marr, 2010; Llorens-Martin, 2018). In humans, long-term exercise improves blood flow while also increasing hippocampal volume and neurogenesis in subjects with AD (Karssemeijer et al., 2017; Meng et al., 2020). AHN is also altered in transgenic mouse models of AD (Jin et al., 2004; Wen et al., 2004; Kuhn et al., 2007; Taniuchi et al., 2007; Verret et al., 2007; Niidome et al., 2008; Yu et al., 2009; Chuang, 2010; Demars et al., 2010; Elder et al., 2010; Krezymon et al., 2013; Unger et al., 2016), and exercise has been reported to improve learning behavior in some of these models. For example, in *APP*^*KM*670/671*NL*^*/PSEN1*^Δ*exon*9^(APP/PS1) transgenic mice, 2 days of treadmill running for 30 min was associated with increased brain activity, as shown by increased theta rhythm on electroencephalography (Borchelt et al., 1996). In the same APP/PS1 model, 20 weeks of treadmill training improved learning behavior and reduced brain levels of the 42-aminoacid form of amyloid beta peptide (Aβ) (Bo et al., 2014). In the 5xFAD mouse model of AD, 4 months of voluntary PE in a running wheel improved learning behavior and increased levels of BDNF and synaptic markers in the hippocampus (Choi et al., 2018).

BCI-838 (MGS0210) is a pro-drug that is metabolized to generate a negative allosteric modulator that targets group II metabotropic glutamate receptors (mGluR2/3) (Kim et al., 2014; Perez-Garcia et al., 2018). BCI-838 is metabolized in the liver into BCI-632, which is the active compound delivered to the brain. As a class, mGluR2/3 receptor antagonists are proneurogenic, as evidenced by their stimulation of AHN while also enhancing learning behavior and exerting broad anxiolytic and antidepressant properties (Chaki and Fukumoto, 2018). Previously, BCI-838 administration was observed to improve learning behavior and reduce anxiety in a transgenic mouse model of cerebral amyloidosis (Kim et al., 2014) and in a rat model of blast-related traumatic brain injury (Perez-Garcia et al., 2018).

Since both BCI-838 and PE are associated with enhanced AHN, we sought to determine whether BCI-838 might mimic the effects of PE, and whether a combined treatment with both drug and PE might be additive or synergistic. Our findings demonstrate that BCI-838 stimulates AHN, improves recognition memory, and upregulates BDNF and PIK3C2A levels of the mammalian target of rapamycin (mTOR) pathway, as well as metabotropic glutamate receptors in APP/PS1 transgenic mice, thereby mimicking some of the effects of PE.

## Materials and methods

### Animals

**Figure 1:**
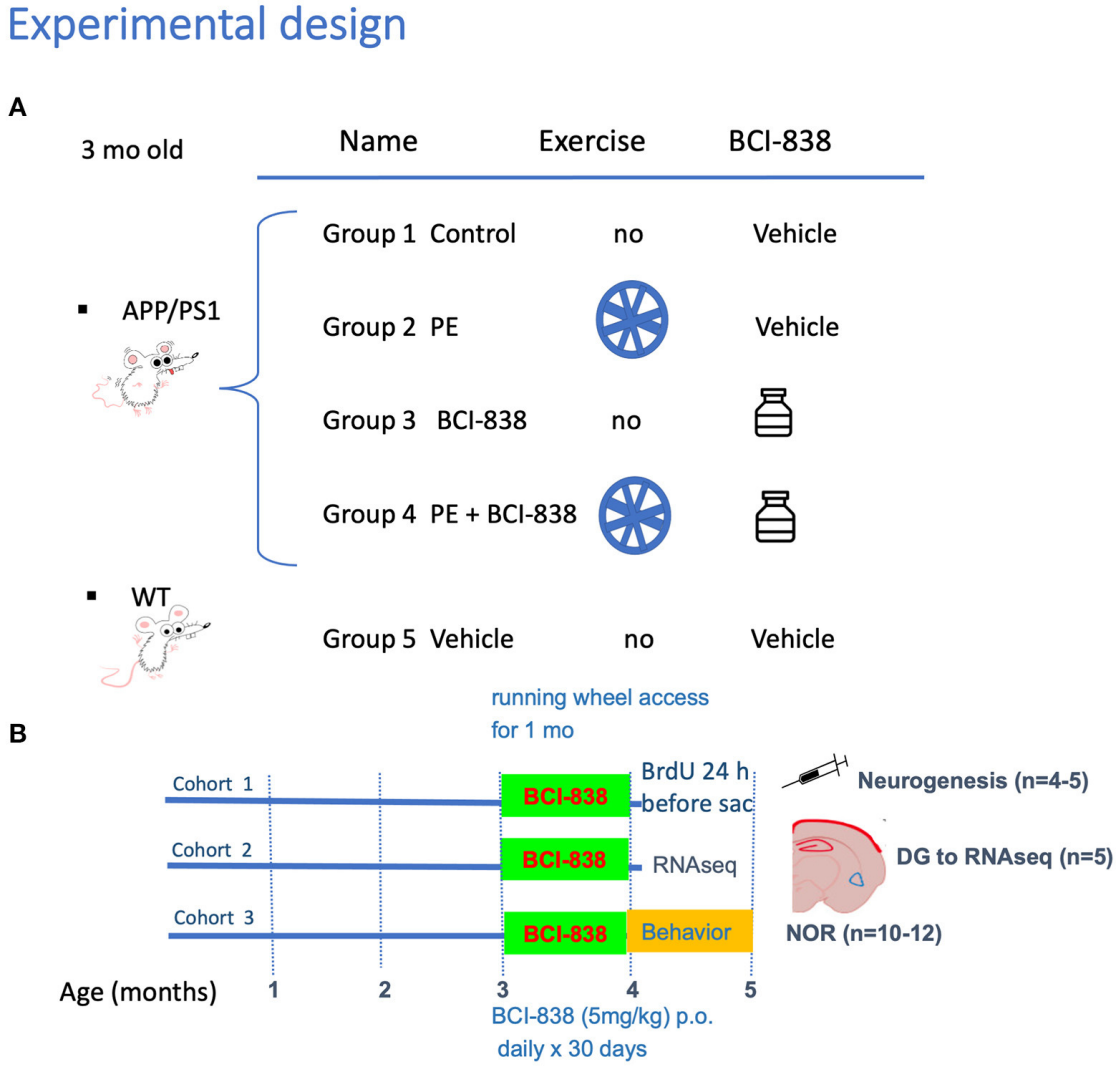
Experimental design and spontaneous activity. **(A)** APP/PS1 mice were divided into four groups: Group 1 no PE/no drug; Group 2 PE/no drug; Group 3, no PE/drug; and Group 4, PE + drug. Group 5 consisted of WT mice (C57BL/6). **(B)** Groups receiving PE were given *ad libitum* access to a running wheel and treatment with drug or vehicle was continued for 30 days. All studies used male mice and included three cohorts. The first cohort was processed for neurogenesis studies, following perfusion-fixation with PFA 4% (five animals per group). The second cohort was processed for RNA and biochemical studies. We sacrificed animals by CO_2_ narcosis and the DG was removed for RNAseq and determination of BDNF by qPCR. We processed five animals per group. The third cohort was used for behavior testing (10-12 mice per group).

Experimental procedures were conducted in accordance with and approved by the Institutional Animal Care and Use Committee (IACUC) of the James J. Peters VA Medical Center. Studies were conducted in compliance with the US Public Health Service policy on the humane care and use of laboratory animals, the NIH Guide for the Care and Use of Laboratory Animals, and all applicable Federal regulations governing the protection of animals in research. APP^*KM*670/671*NL*^/PSEN1^Δ*exon*9^ (APP/PS1) transgenic mice (Cat# 34832-JAX; RRID: MMRRC_034832-JAX) were obtained from Jackson Laboratories (JAX). Mice used for this study were generated by breeding APP/PS1 mice with C57BL/6 wild-type (WT) mice (also obtained from JAX; Cat#000664 RRID: IMSR_JAX: 000664). Non-transgenic littermates were used as wild type controls and all mice were on a C57BL/6 background. All studies used male mice and included three cohorts. Figure 1 outlines the studies and how the mice were distributed across the different assays. The first cohort was perfusion-fixed with 4% paraformaldehyde for neurogenesis studies (five animals per group). The second cohort was processed for biochemical studies. These mice were euthanized by CO_2_ narcosis, and the brain was divided into 2 hemispheres with the left dentate gyrus (DG) used for RNAseq and the right DG for ELISA to measure BDNF levels. The third cohort used 10 to 12 mice per group for behavioral testing. To avoid the confounding effects of behavioral testing, tissue from mice that underwent behavioral testing was not used for any of the RNA or neurogenesis studies. The total number of mice used for all studies was 105.

### Physical exercise exposure

Cages were fitted with running wheels obtained from Columbus Instruments and equipped with Activity Wheel Monitoring Software. Mice were singly housed with one running wheel per cage and were allowed access to running wheels *ad libitum*. Running wheel (Keiser et al., 2009) data (number of wheel revolutions) were recorded. Cages that housed mice in the non-exercise groups were fitted with dummy wheels that the mice could enter but could not run.

### Drug administration

BCI-838 was prepared as previously described (Perez-Garcia et al., 2018). BCI-838 was dissolved in a solution of 5% carboxymethylcellulose (CMC; Sigma Aldrich) and 0.3% 2N hydrochloric acid solution (Sigma Aldrich). Animals were divided into five experimental groups: (1) APP/PS1 mice treated with vehicle (APP/PS1 control); (2) APP/PS1 mice treated with vehicle and exposed to running wheels (APP/PS1 + PE); (3) APP/PS1 mice treated with 5 mg/kg BCI-838 (APP/PS1 + BCI-838); and (4) APP/PS1 mice treated with 5 mg/kg BCI-838 and exposed to running wheels (APP/PS1 + BCI-838 + PE combination) and (5) WT mice treated with vehicle (Figures 1A, B). BCI-838 was administered daily for 30 days by oral gavage using a 5-cm straight stainless-steel gavage needle with a 2-mm ball tip (Fischer Scientific).

### Bromodeoxyuridine injections

All animals received a single intraperitoneal injection of BrdU (50 mg/kg of body weight) 24 h before sacrifice at the end of drug treatment. BrdU (Sigma) was dissolved in saline solution (0.9% NaCl in sterile H_2_O) warmed to 40^o^C and gently vortexed. The solution was allowed to cool to room temperature (25^o^C) and injected 24 h before sacrifice.

### Behavioral testing

Behavioral tests began at the end of the 30 days of drug administration for the third cohort (Figure 1). Each group included 10 to 12 mice. All behavior sessions and trials were recorded by video camera (Sentech) and analyzed with ANYMAZE software (San Diego Instruments).

### Novel object recognition

Mice were habituated to the circular arena (30 cm in diameter length × 30 cm height) for 10 min, 24 h before training. On the training day, two identical objects were placed in opposite ends of the empty arena, and the mouse was allowed to freely explore the objects (Ob1 and Ob2) for 5 min. After 24 h, during which the mouse was held in its home cage, one of the two now familiar objects (FO) was replaced with a novel object. The mouse was allowed to freely explore the FO and NO for 5 min to assess long-term memory (LTM). Raw exploration times for each object were expressed in seconds. Object exploration was defined as sniffing or touching the object with the nose placed at < 2 cm. Offline analysis by an investigator blind to the treatment status of the animals was performed. During the training session, two identical objects made of LEGO plastic material (≈2.5 cm diameter and height) were used. In the long-term memory (LTM) testing, one of the LEGOs was replaced with a ceramic cup (≈2.5 cm maximum diameter and height). No animals were observed to climb the objects. All objects were wiped with 70% ethanol between trials.

### Tissue processing and immunohistochemistry

Animals were sacrificed at the conclusion of the drug treatment, PE exposure, and behavioral testing. We induced deep anesthesia with a solution of 100 mg/kg ketamine and 20 mg/kg xylazine, and mice were euthanized by transcardial perfusion with cold PBS (20 ml) followed by perfusion with 4% paraformaldehyde (PFA) in PBS. After perfusion, brains were removed and postfixed in PFA 4% for 48 h, transferred to PBS, and stored at 4^o^C until sectioning. Coronal sections (50 μm) were cut through the entire extent of the hippocampus using a Leica VT1000 S Vibratome (Leica). The sections were stored at −20^o^C in a cryoprotectant solution (25% ethylene glycol and 25% glycerine in 0.05 M PBS) until processing for immunofluorescence. Stereology-based counting was performed as described (Perez-Garcia et al., 2016). Every sixth section in a series through the hippocampus was processed for immunohistochemistry so that the interval between sections within a given series was 300 μm. BrdU and doublecortin (DCX) stainings were performed as previously described in Perez-Garcia et al. (2016, 2018). 4-5 animals per group were included in the analyses.

### RNA extraction and BDNF levels

DG samples were homogenized in QIAzol Lysis Reagent (Qiagen), and total RNA purification was performed with the miRNeasy Micro kit (Qiagen). BDNF mRNA levels were determined by real-time quantitative PCR (qPCR). 200 ng of total RNAs were reverse transcribed using the High-Capacity RNA-to-cDNA Kit (Applied Biosystem). cDNAs were subjected to real-time qPCR in a StepOne Plus system (Applied Biosystem) using the TaqMan Gene Expression Master Mix (Applied Biosystem). qPCR consisted of 40 cycles, 10 s at 95^o^C, 20 s at 60^o^C, and 15 s at 70^o^C each. Ct values were normalized to the expression level of GAPDH. Five animals per group were included in the analyses. Sample sizes were selected based on previous studies (Readhead et al., 2018).

### Statistical analyses

Values are expressed as mean ± SEM. For behavioral analysis, stereology, spontaneous activity analysis, biochemical analysis, and stereological analysis, statistical tests were performed using unpaired *t*-tests, one-way ANOVA, repeated measures ANOVA, and the Kuskall–Wallis non-parametric test following by an uncorrected Dunn test. When more than two groups were compared, a one-way ANOVA was follow by Sidak's post-test if only selected groups were compared or Tukey's multiple comparison's test if all groups were compared. When repeated-measures ANOVA was used, sphericity was assessed using Mauchly's test. If the assumption of sphericity was violated (*p* < 0.05, Mauchly's test), significance was determined using the Greenhouse-Geisser correction. Pearson correlation coefficients were calculated. Statistical analysis was performed using Prism 9.4.1 (GraphPad Software) and SPSS v27.0.1.0 (IBM).

### RNA sequencing

Total RNA from DG of all mice was subjected to RNA sequencing. The sequencing library was prepared using the NEB library prep kit (Novogene). Five animals per group were included in the analyses based on the experience of the Gandy/Ehrlich lab in previous studies (Readhead et al., 2016).

### Read alignment and gene expression counts

Computational analysis was performed using R version 4.0.2 (r-project.org). Paired-end RNA-Seq fastq files for 24 samples were aligned to the Mouse Reference genome (mm10) using the STAR (Dobin et al., 2013) read aligner. As part of quality control and to allow discrimination between human APP and PSEN1 transgenes and mouse APP and PSEN1 genes, all samples were also aligned to *Homo sapiens* reference genome (Grch38), and corresponding gene expressions were checked for sanity. Mapped reads were summarized to gene counts using the subread function of featureCounts (Liao et al., 2014).

### Quality control

Variance partitioning performed using *variancePartition* (Hoffman and Schadt, 2016) R package showed a high fraction of unexplained variance (“residuals,” see Supplementary Figure 1). We addressed this by applying surrogate variable analysis (SVA) using the *sva* (Leek et al., 2012) R package, to estimate unwanted sources of variation. The svaseq function estimated five surrogate variables (*n*.sv = 5), which were then included in our differential expression analysis as covariates to be accounted.

### Differential expression analysis

Gene count matrices were generated separately for five groups of 24 samples in total: WT (*n* = 5), APP/PS1 control (*n* = 5), APP/PS1 + BCI-838 (*n* = 5), APP/PS1 + PE (*n* = 4), APP/PS1 combination (*n* = 5); and six primary comparisons: APP/PS1 + BCI-838 vs. APP/PS1 control, APP/PS1 + PE vs. APP/PS1 control, APP/PS1 combination vs. APP/PS1 control, APP/PS1 combination vs. APP/PS1 + BCI-838, APP/PS1 combination vs. APP/PS1 + PE, APP/PS1 + BCI-838 vs. APP/PS1 + PE, and APP/PS1 control vs. WT. The overall gene count matrix was corrected for library size, normalized, and the resulting 18,357 genes were statistically analyzed for differentially expressed genes (DEGs) using DESeq2 (Love et al., 2014). *p*-values were adjusted using the Benjamini-Hochberg method, where DEGs were defined at a false discovery rate (FDR) of 0.05. DEGs sorted by adjusted *p*-values are provided in Supplementary Table 1.

### Gene set enrichment analysis

DEG sets per primary comparison were tested for statistical enrichment using the EnrichR (Kuleshov et al., 2016) R package for transcription factors, pathways, and gene ontology against 11 relevant public databases. Relevant matches with an FDR of 0.05 were identified. Enrichments are provided in Supplementary Table 2.

### Code availability

All scripts used for analysis are publicly available via our GitHub pages: https://github.com/MesudeBicak/AlzheimersDiseaseResearch/tree/master/BCI838vsExerciseAPPPS1Mice.

### Computational drug repurposing and chemogenomic enrichment analysis

Drug-induced gene expression fold-changes were obtained from the Connectivity Map database (Lamb et al., 2006). Individual expression profiles (6,100) were merged into a single representative signature for 1,309 unique compounds, according to the prototype-ranked list method (Iorio et al., 2010). Each compound was scored according to the transcriptomic similarity with BCI-838 transcriptomics (DEGs from APP/PS1 + BCI-838 vs. APP/PS1). Compounds were ranked in order of descending connectivity score. For each compound in the drug signature library, referenced drug–target associations (Law et al., 2014), predicted off-targeting (Keiser et al., 2009), and side effects were collected. For each of these features, we calculated a running sum enrichment score, reflecting whether that feature was over-represented among the compounds with transcriptomic similarity to BCI-838. Two-tailed *p*-values were based on comparison with 10,000 permuted null scores, generated from randomized drug target sets that contain an equivalent number of compounds to the true set under evaluation, and adjusted using the Benjamin–Hochberg method. Computational screening and chemogenomic enrichment analysis were performed using R. Drug repurposing results are provided in Supplementary Table 3.

## Results

### Experimental design for exposure of APP/PS1 mice to PE and treatment with BCI-838

**Figure 2:**
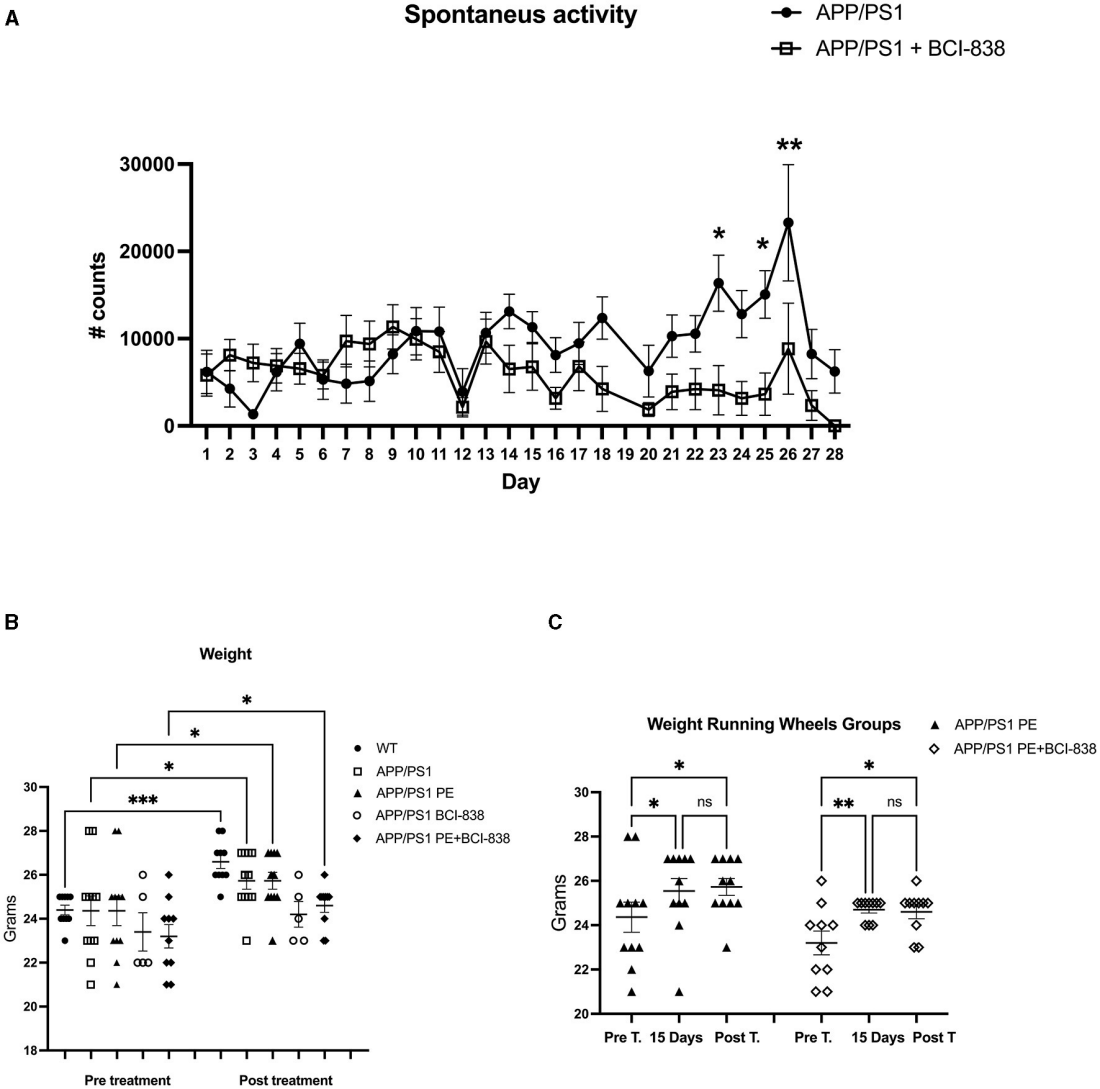
BCI-838 decreased spontaneous activity after 2 weeks of treatment. **(A)** Number of running wheel turns (# counts) are shown for the two groups that received running wheel access. APP/PS1 mice treated with BCI-838 decreased running wheel use during the last 2 weeks of drug treatment and running wheel exposure. Repeated measures ANOVA over the entire 30 days of treatment revealed a significant difference in running wheel activity within groups (*F*_3.8,61.1_ = 3.695, *p* = 0.011) but no day*condition interaction effect (*p* = 0.052). A test of between subject effects over the 30 days revealed no significant effect of condition (*F*_1,16_ = 2.399, *p* = 0.14). Tests of within subject effects revealed significant effects of running wheel activity if analyzed over 1–14 days (*F*_3.5,59.5_ = 3.579, *p* = 0.014 for activity, *F*_3.5,59.5_ = 2.062, *p* = 0.105 for day*condition) and 14–28 days (*F*_2.9,47.0_ = 4.414, *p* = 0.008 for activity, *F*_2.9,47.0_ = 4.414, *p* = 0.366 for day*condition). There was no difference in running wheel activity between groups over days 1–14 (*F*_1, 17_ = 0.799) whereas activity was reduced in mice that received PE + drug between days 14–28 (*F*_1,16_ = 6.052, *p* = 0.026). Values significantly different between groups at individual time points are indicated by asterisks (*p < 0.05, **p < 0.01, unpaired *t*-tests). Values are expressed as mean ± SEM. 12 mice per group for each cohort were used. **(B)** Weights of mice before and after treatment with PE and/or BCI-838. There were no differences between groups analyzed pre-treatment (one way ANOVA, *F*_4,42_ = 0.9107, *p* = 0.4666), but differences between WT and BCI-838 and PE + BCI-838 group were found post-treatment (*F*_4,42_ = 5.588, *p* = 0.0011). However, a repeated measures ANOVA comparing pre-treatment to post-treatment revealed that mice gained weight after treatment (*F*_2.575,21.17_ = 8.547, *p* = 0.0010). Asterisks indicate significant between group differences when selected groups were directly compared using Sidak's test (*p < 0.05, **p < 0.01, ***p < 0.001). **(C)** Running wheel activity in APP/PS1mice treated with PE or PE + BCI-838 is shown pre-treatment, after 15 days of treatment and post-treatment. A one-way ANOVA revealed significant between group differences when all 6 groups were compared *F*_(5,57)_ = 3.589, *p* = 0.0069. Asterisks indicate significant between group differences when indicated groups were directly compared using Sidak's test (*p < 0.05, **p < 0.01, ns, not significant).

Mouse groups received treatment with BCI-838 or vehicle for 1 month with or without PE (Figure 1). PE consisted of *ad libitum* access to a running wheel. Three-month-old mice were divided into five experimental groups: (1) APP/PS1 mice treated with vehicle (APP/PS1 control); (2) APP/PS1 mice treated with vehicle and exposed to running wheels (APP/PS1 + PE); (3) APP/PS1 mice treated with 5 mg/kg BCI-838 (APP/PS1 + BCI-838); and (4) APP/PS1 mice treated with 5 mg/kg BCI-838 and exposed to running wheels (APP/PS1 + BCI-838 + PE combination) and (5) WT mice treated with vehicle (Figures 1A, B). In this design, comparison of the single group of untreated wild type mice (Group 5) to untreated APP/PS1 mice (Group 1) served as a positive control for appearance of the transgene-related behavioral phenotype while comparison of the treated groups (2, 3, and 4) to group 1 provided a measure of the effects of the various treatments. Three cohorts of mice were studied. One cohort was used for behavioral analysis, the second for neurogenesis assays and the third for transcriptomic analysis. Spontaneous running wheel activity was recorded in the two cohorts exposed to active running wheels. In these two cohorts, when compared to APP/PS1 control, APP/PS1 + BCI-838 showed decreased spontaneous activity during the last 2 weeks of the treatment protocol, as evidenced by reduced counts of running wheel activity (Figure 2A). Weights of mice before and after treatment are shown in Figure 2B. All groups gained weight during treatment (Figure 2B) with no differences between APP/PS1 mice that received PE alone vs. PE + BCI-838 (Figure 2C).

### Both BCI-838 and PE can improve recognition memory in a novel object recognition test

**Figure 3:**
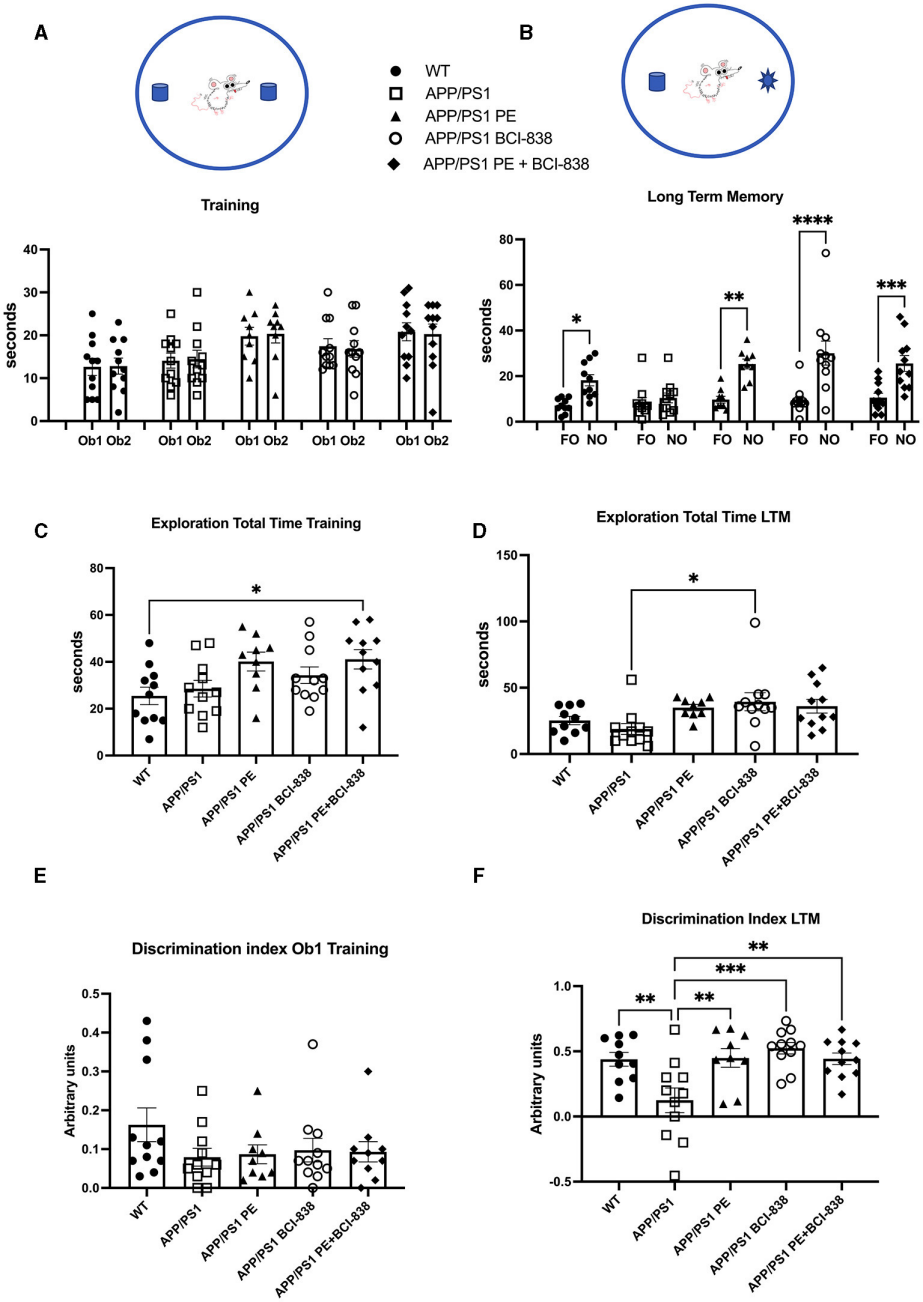
Effect of BCI-838 treatment and PE on novel object recognition (NOR). **(A)** Training. No differences were found among groups during the training when the mice explored the identical objects, Object 1 (Ob1) and Object 2 (Ob2). **(B)** Long-term memory session (LTM). During the LTM testing conducted at 24 h after training, wild type mice spent more time exploring the novel object (NO) as compared to the familiar object (FO). APP/PS1 mice treated with vehicle explored the FO and the NO similar amounts of time whereas APP/PS1 mice exposed to PE, treated with BCI-838 or the combination of BCI-838 + PE for 1 month showed a preference for the NO compared to the FO. Values significantly different are indicated by asterisks (*p < 0.05, **p < 0.01, ***p < 0.001, ****p < 0.0001, ANOVA followed by Sidak's test, comparing selected pairs of columns). **(C)** Total time spent exploring the objects during training is shown. The combination of BCI-838 + PE mice explored the objects more compared to WT mice (one-way ANOVA: *F*_4,48_ = 3.286, *p* = 0.0185; *p < 0.05, Tukey's multiple comparisons test). **(D)** During LTM testing, APP/PS1 mice treated with BCI-838 alone were more exploratory than APP/PS1 mice treated with vehicle (*F*_2,47_ = 3.308, *p* = 0.0181; *p < 0.05, Tukey's multiple comparisons test). **(E)** A discrimination index calculated for the training session showed no difference in object preference between groups (*F*_4, 47_ = 1.226, *p* = 0.3124). **(F)** A discrimination index calculated for LTM (*F*_4,47_ = 6.131, *p* = 0.0005) found differences between APP/PS1 treated with vehicle and all other groups but no differences between treatments (** *p* < 0.01, ***p < 0.0001, Tukey's multiple comparisons test). Values are expressed as mean ± SEM). Ten to 12 mice per group were used for each cohort.

We probed the effect of PE, BCI-838, or the two combined, on recognition memory in APP/PS1 mice (Figure 3). Novel object recognition (NOR) is a standard behavioral test used to evaluate hippocampal- and perirhinal-dependent memory. NOR is widely used to assess the progression of behavioral deficits in mouse models of AD. During training, no differences in object preference were observed among groups (Figure 3A). During LTM testing (Figure 3B), WT mice spent more time exploring the NO than the FO (*p* = 0.028). APP/PS1 mice explored the FO and NO for the same amount of time (*p* = 0.989), consistent with the conclusion that APP/PS1 mice at 4 months of age have impaired recognition memory. However, APP/PS1 mice exposed to 1 month of PE spent more time exploring the NO when tested 24 h after training (*p* = 0.0013). APP/PS1 mice treated with BCI-838 for 1 month also spent more time exploring the NO (*p* = 0.0001). APP/PS1 mice treated with the combination of BCI-838 and PE for 1 month also showed a preference for the NO compared to FO (*p* = 0.0006). Total exploration times between groups during training differed only in that APP/PS1 mice treated with PE and BCI-838 spent more time exploring the objects than wild type mice administered vehicle (Figure 3C) while in LTM testing APP/PS1 mice treated with BCI-838 alone were more exploratory than APP/PS1 mice treated with vehicle (Figure 3D). A discrimination index calculated for the training session showed no differences in object preference between the groups (Figure 3E) and one calculated for the LTM (Figure 3F), confirmed conclusions drawn from the raw object exploration data (Figure 3B) suggesting that recognition memory had been rescued in all treated groups compared to APP/PS1 mice administered vehicle alone. These results suggest that 1-month of either PE or BCI-838 drug administration, as well as the combination of both, can reverse impaired recognition memory in APP/PS1 mice.

### BCI-838 administration, alone or in combination with PE, enhances hippocampal neurogenesis

**Figure 4:**
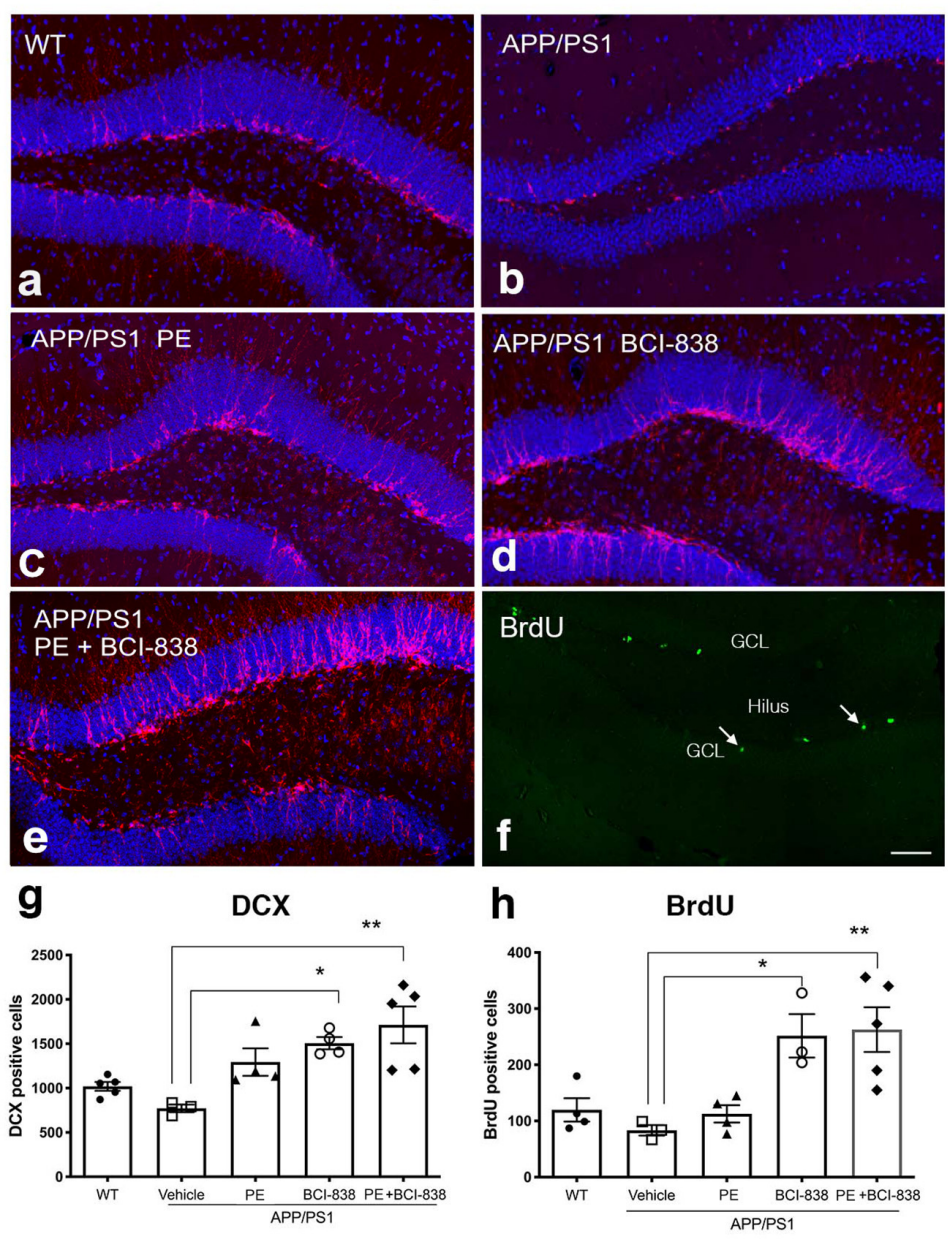
Quantification of neurogenesis in WT, APP/PS1 treated with vehicle, PE, BCI-838 or the combination. **(a–e)** Images of DCX staining in WT, APP/PS1, APP/PS1 + PE, APP/PS1 + BCI-838, and APP/PS1 combination. **(f)** Representative image of BrdU staining in a WT mouse. The hilus and granule cell layer (GCL) are indicated. Arrows mark BrdU-labeled cells in the subgranular zone. Scale bar 50 μm. **(g)** Total number of doublecortin-labeled cells was increased in the APP/PS1 + BCI-838 and APP/PS1 combination as compared to APP/PS1 control. Values are expressed as mean ± SEM and differences among groups are indicated by asterisks (*p < 0.05, **p < 0.01, one-way ANOVA followed by Tukey's test). **(h)** Total number of BrdU-labeled cells was increased in APP/PS1 + BCI-838 and APP/PS1 combination compared to APP/PS1 control. Values significantly different among groups are indicated by asterisks as in panel **(g)**. 4–5 mice per group for each cohort were used.

We next probed the effect of PE, BCI-838 administration, or the combination of both on AHN in APP/PS1 mice (Figure 4). Using immunofluorescence staining, we quantified newly generated neurons (doublecortin-labeled; DCX) cells (Figures 4a–e) and newly generated cells (BrdU-labeled; Figure 4f) in APP/PS1 mice treated with vehicle or PE, BCI-838, or both. While the number of DCX-labeled neurons was unchanged in APP/PS1 mice treated with vehicle vs. WT mice, this number was increased in APP/PS1 mice treated with BCI-838 or with BCI-838 plus PE, when either was compared to APP/PS1 treated with vehicle (*F*_4,16_ = 1.367, *p* = 0.0025; *p* < 0.05, ANOVA Tukey's multiple comparisons Figures 4a–e, g). Moreover, we observed that the total number of BrdU-labeled cells was increased in APP/PS1 mice treated with BCI-838 or BCI-838 plus PE as compared to APP/PS1 mice treated with vehicle (*F*_4,14_ = 1.734; *p* = 0.0148, *p* < 0.05, ANOVA Tukey's multiple comparisons Figures 4f, h). By contrast, PE alone did not increase the number of DCX or BrdU-labeled cells compared to vehicle treated WT or APP/PS1 mice.

### Transcriptomic profiles from the hippocampal DG of APP/PS1 mice show that BCI-838 affects both exercise-related and exercise-independent molecular pathways

**Figure 5:**
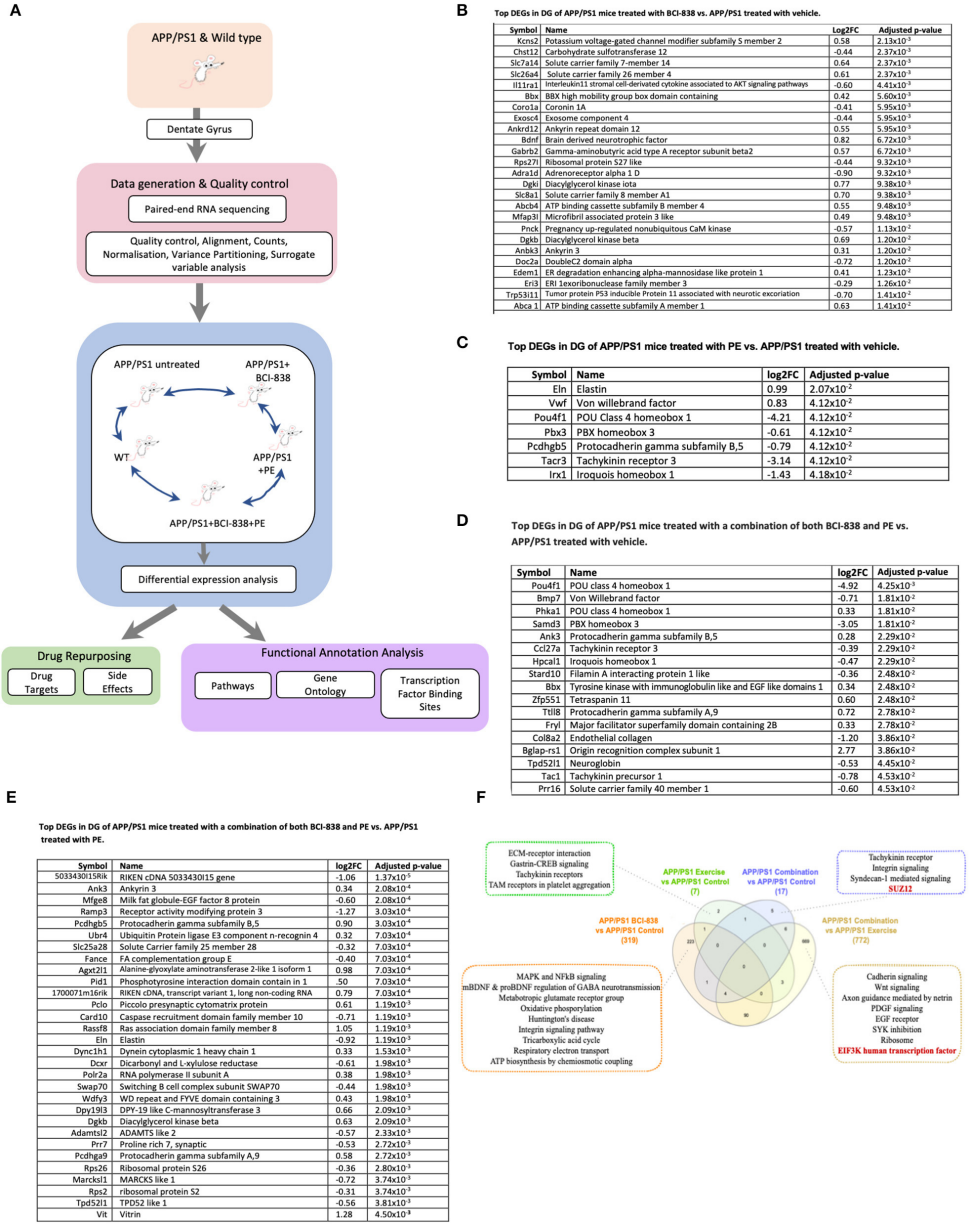
Differential gene expression and enrichment analysis summary in dentate gyrus (DG) of APP/PS1 mice treated with BCI-838, PE or a combination of both BCI-838 + PE. RNA sequencing was performed on dentate gyrus for five groups of four-month-old APP/PS1 and WT mice, comprising a total of 24 samples. **(A)** Schematic overview of mouse AD transcriptome analysis. **(B)** Top DEGs in DG of APP/PS1 mice treated with BCI-838 vs. APP/PS1 treated with vehicle. **(C)** Top DEGs in DG of APP/PS1 mice treated with PE vs. APP/PS1 treated with vehicle. **(D)** Top DEGs in DG of APP/PS1 mice treated with a combination of both BCI-838 and PE vs. APP/PS1 treated with vehicle. **(E)** Top DEGs in DG of APP/PS1 mice treated with a combination of both BCI-838 and PE vs. APP/PS1 treated with PE. **(F)** Venn Diagram of DEGs and selected pathway enrichments of known or suspected relevance to AD pathophysiology, shared across different comparisons of APP/PS1 mice groups (transcription factor enrichments shown in red).

We generated transcriptomic profiles from all four groups of four-month-old APP/PS1 mice and one group of WT mice and performed RNA sequencing on 24 DG samples (Figure 5). When we performed variance partitioning analysis as part of quality control, we noticed a high fraction of unexplained variance in our RNA-Seq dataset and used surrogate variable analysis to address this issue (see Methods and Supplementary Figure 1). We then performed DEG analysis to compare all mice groups with each other, from APP/PS1 mice exposed to either BCI-838, PE or a combination, as well as WT mice. DEGs were identified at a false discovery rate (FDR) of 0.05 (see Supplementary Table 1 for a list of all DEGs for all comparisons). To investigate biological pathways that might be differentially dysregulated by administration of BCI-838 or exposure to exercise, we performed gene set enrichment analysis (GSEA) on the identified DEGs, resulting in enriched biological pathways, gene ontology sets, and transcription factor binding sites at an adjusted *p*-value < 0.05 (see Supplementary Table 2 for GSEA results). Top DEGs and GSEA results sorted by their adjusted *p*-values are summarized in Figure 5.

The most striking changes across all groups were the transcriptomic changes induced by one month of BCI-838 administration in the DG of APP/PS1 (APP/PS1 + BCI-838), which identified 319 DEGs. One hundred ninety-one up-regulated DEGs from this analysis included BDNF, as well as PIK3C2A of the PI3K-mTOR pathway, whereas 128 down-regulated DEGs included IL11RA1, the interleukin 11 receptor of the AKT signaling pathway, as well as EIF5A, the eukaryotic translation initiation factor. Up-regulated DEGs also included ITGB8 of the integrin family, an extracellular matrix (ECM) receptor, known to regulate neurogenesis and neurovascular homeostasis in adult brain (Mobley et al., 2009). Of further relevance, dysregulation of several GABA (inhibitory) receptors, GABRB1, GABRB2, and GABRD, and up-regulation of two metabotropic glutamate receptor subunits, GRM1 and GRM5, and the ATP- binding AD risk gene ABCA1 (Lupton et al., 2014) were identified among DEGs. GSEA pointed to SP1 as the top transcription factor binding site for down-regulated DEGs; other differentially regulated pathways included neurodegenerative diseases, such as AD and Huntington disease, whereas up-regulated DEGs were found enriched for glutamate receptor activity and AKT1 knockdown pathways as expected.

After 1 month of PE, the transcriptomes of the hippocampal DG of APP/PS1 mice (APP/PS1 + PE) were compared to those of APP/PS1 control mice, leading to the identification of seven DEGs (two up-regulated, five down-regulated) that were found to be enriched for transcripts associated with “response to mTOR inhibitor,” “extracellular matrix receptor reaction,” “trigeminal nerve development,” “peripheral nervous system neuron development,” and “polycomb repressive complex component SUZ12,” which plays a critical role in regulating neurogenic potential and differentiation of embryonic stem cells (Pasini et al., 2007). Meanwhile, compared with untreated APP/PS1 mice, in APP/PS1 mice treated with the combination of PE and BCI-838 (APP/PS1 combination) for 1 month, 17 DEGs were identified (seven up-regulated, 10 down-regulated), which were enriched for SUZ12, as well as glycogen metabolism and exercise-induced myalgia pathways. When APP/PS1 combination was compared with APP/PS1 + BCI-838, we did not observe any DEGs. However, when APP/PS1 combination was compared with APP/PS1 + PE, we identified 772 DEGs, which were found to be enriched in EIF3K and REST transcription factors, both shown to regulate the mTOR signaling pathway in adipocyte yeast (Harris et al., 2006) and oral cancer cells (Cho et al., 2015), respectively. Cadherin signaling, axon guidance mediated by netrin, epidermal growth factor (EGF) receptor signaling, SYK inhibition and knockdown were also among differential pathways of relevance with strong associations to mTOR activity and signaling, suggesting neurogenesis and PE, thereby demonstrating the validity of our RNA-Seq data and experiment.

Finally, a comparison of the transcriptomes of the hippocampal DG of APP/PS1 control mice against those of WT mice showed 60 DEGs (41 upregulated, 19 down-regulated) found to be enriched in calcium cation antiporter activity and ATP-dependent microtubule motor activity pathways, in line with highlighted findings from our reported transcriptomic analysis of APP/PS1 vs. WT (Readhead et al., 2016).

### BDNF levels in the hippocampal DG of APP/PS1 mice following BCI-838 treatment

**Figure 6:**
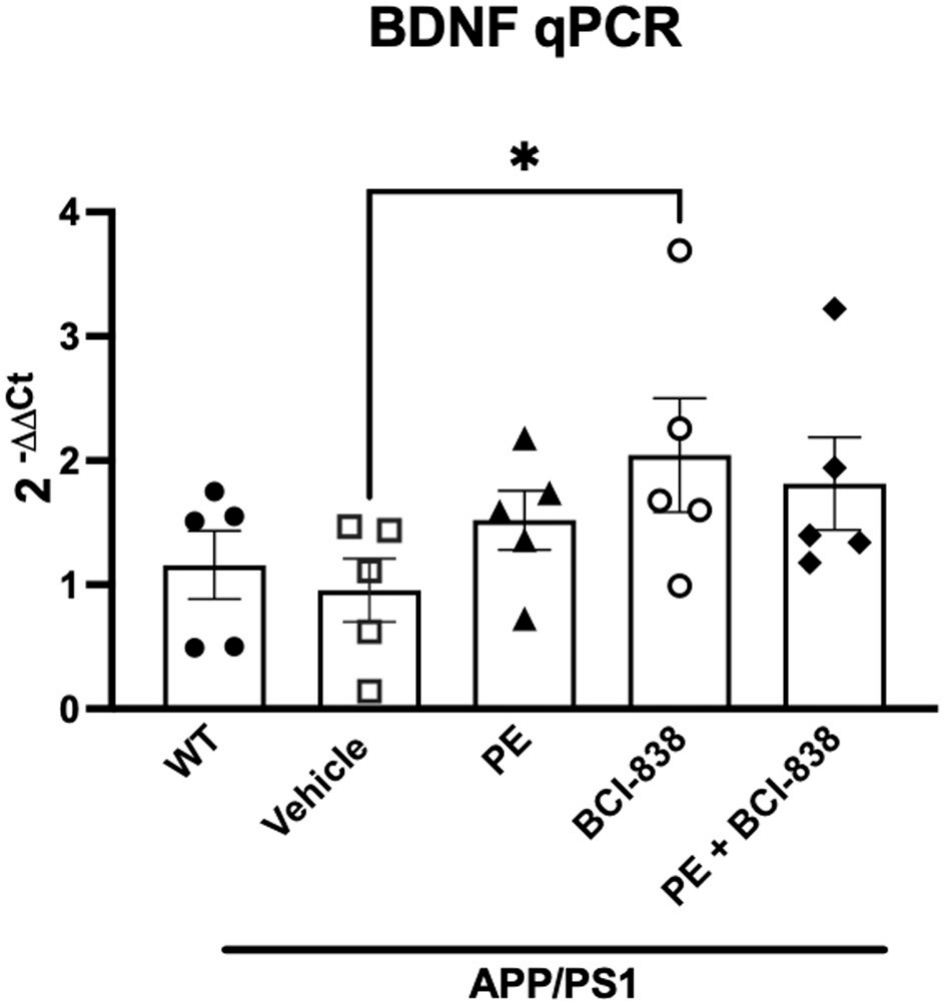
BDNF analysis in APP/PS1 mice treated with BCI-838 or PE. qPCR for BDNF mRNA. Asterisk indicates *p* = 0.025 (Kuskall–Wallis, uncorrected Dunn test). Five mice per group were used for the analysis.

Increased levels of BDNF have been implicated in the beneficial effects of PE in 5xFAD transgenic mice (Choi et al., 2018). Figure 6 shows BDNF RNA levels in mice following treatment with PE and BCI-838. A one-way ANOVA did not indicate between group differences in BDNF RNA levels. Only if a Kuskall–Wallis non-parametric test, followed by an uncorrected Dunn test, were used were BDNF mRNA levels found to be significantly increased in APP/PS1 mice treated with BCI-838, but not in APP/PS1 mice treated with PE (Figure 6, *p* = 0.0255, Kuskall–Wallis).

### Transcriptomically similar drug targets to BCI-838 are enriched for glutamate receptor targets, restore adult neurogenesis, and improve hippocampal memory

**Figure 7:**
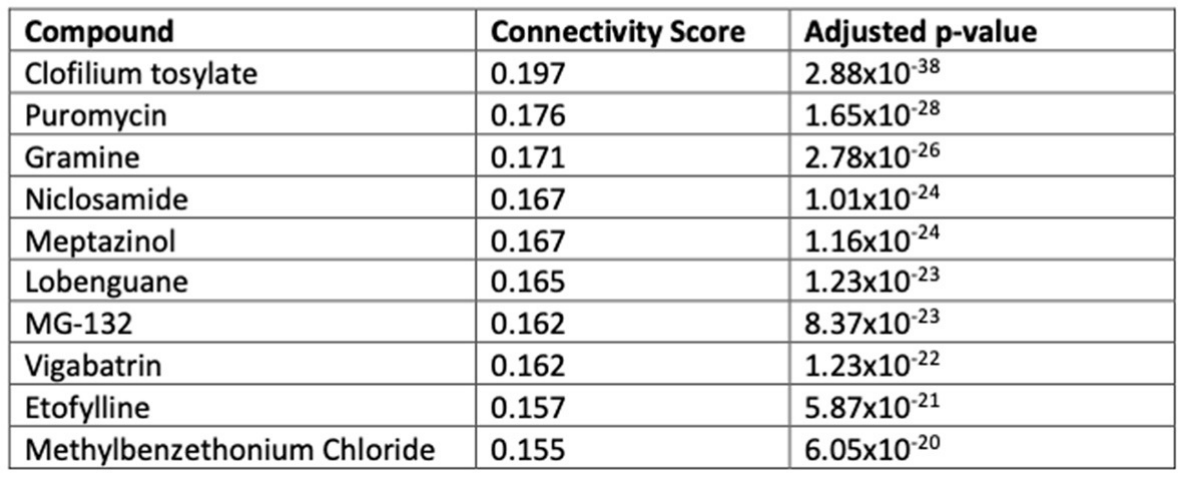
Top drugs similar to BCI-838 based on transcriptomic activity.

**Figure 8:**
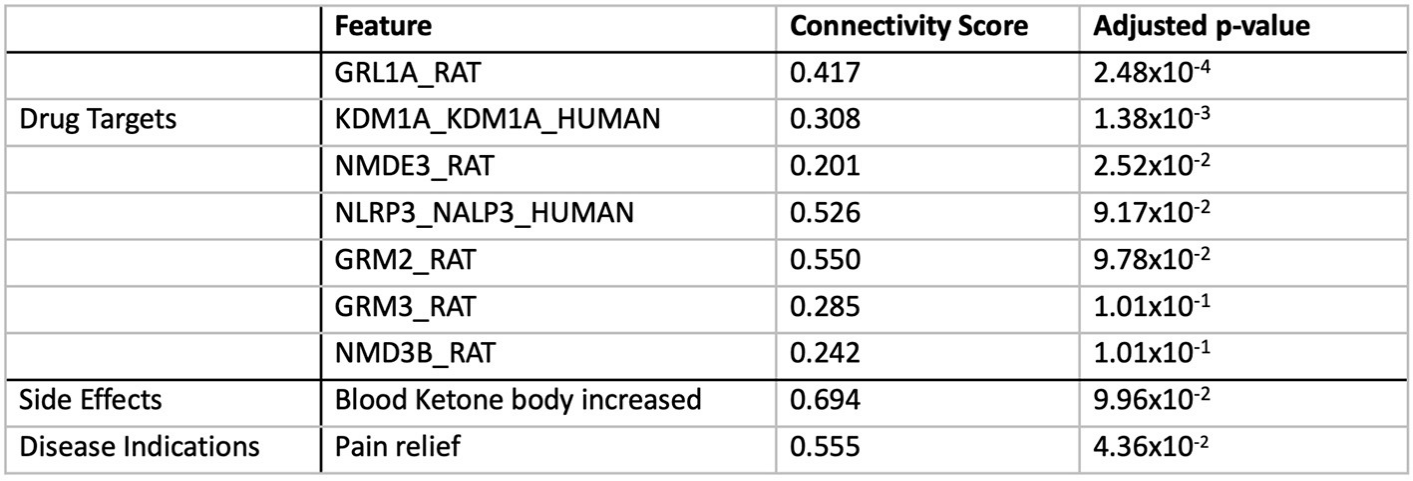
Drug targets and side effects enriched among compounds with transcriptomic similarity to BCI-838.

We performed computational drug repurposing to identify compounds that induce a transcriptomically similar profile to BCI-838 using a modified Connectivity Mapping (Lamb et al., 2006) approach (Figure 7). We then performed a chemogenomic enrichment analysis (Readhead et al., 2018) on these compounds to identify drug targets and side effects that were enriched among compounds that were transcriptomically similar to BCI-838 (see Supplementary Table 3 for drug repurposing results). We observed enrichment for protein targets that included several glutamate receptor targets, including GRL1A, NMDE3, GRM2, GRM3, and NMD3B. We also found transcriptomic similarity with other drug targets, such as KDM1A [whose inhibition has been shown to restore adult neurogenesis and improve hippocampal dependent memory (Zhang et al., 2021)] and NLRP3 [which plays a role in regulation of inflammation, immune response, and apoptosis (Hirota et al., 2011)]. We also observed that drugs that were transcriptomically similar to BCI-838 were enriched for the side effect of “increasing blood ketones” as well as associated with “pain relief” (Figure 8). A ketogenic diet has been shown to inhibit the mTOR pathway (McDaniel et al., 2011). Glutamatergic neurotransmission plays a major role in pain sensation and transmission from peripheral tissues into the CNS (Wozniak et al., 2012). These observations comport with findings from DEG and pathway analysis and may suggest follow-up experiments to look for ketogenesis among BCI-838-treated mice.

## Discussion

Metabotropic glutamate receptors (mGluRs) play important roles in regulating glutamatergic neurotransmission. Agents that modulate mGluR2/3 activity have gained attention as possible therapeutic agents for a range of mental health-related disorders (Chaki, 2019). BCI-838 is an mGluR2/3 antagonist pro-drug that is metabolized in the liver into the active metabolite BCI-632. We previously reported that BCI-838 administration reduced anxiety and improved learning behavior in a mouse model of accumulation of oligomeric Aβ^E22Q^ (Kim et al., 2014). More recently, we showed that BCI-838 treatment reversed anxiety- and post-traumatic stress disorder-related traits in a rat model of mild blast injury (Perez-Garcia et al., 2018). In both studies, BCI-838 treatment increased brain levels of markers of neurogenesis (BrdU, DCX, PCNA), reduced anxiety-related behaviors, and reversed learning behavior deficits.

Here we examined the effects of BCI-838, PE, or the combination, in an additional AD mouse model (APP/PS1) that exhibits both oligomeric and fibrillar amyloid pathology. We show that administration of BCI-838 alone or in combination with PE led to stimulation of AHN and improvement in recognition memory. Furthermore, transcriptomic analysis of the hippocampal dentate gyrus following BCI-838 treatment showed up-regulation of BDNF, metabotropic glutamate receptors, and effects on modulators of the PI3K-mTOR pathway. The only general effect of BCI-838 on behavior was that the drug reduced running activity after 2 weeks of treatment, an effect that does not seem to have been previously described for BCI-838 or other compounds in this drug class. It is unlikely however that reduced motor activity in BCI-838 treated mice impacted behavioral test results since exploratory behavior in NOR testing increased in BCI-838 treated mice.

Like BCI-838, PE stimulates neurogenesis in the hippocampal DG (Liu and Nusslock, 2018). Another purpose of the current study was to investigate whether BCI-838 treatment combined with PE could have synergistic effects on recognition memory and AHN in APP/PS1 transgenic mice. Both BCI-838 and PE were associated with restored recognition memory in APP/PS1 mice in a NOR test which is dependent on hippocampal and perirhinal function.

While it is tempting to draw direct comparisons between AD mouse models and human AD, significant differences seem to exist. APP/PS1 mice develop age related amyloid plaque deposition (Borchelt et al., 1996). However, unlike humans, transgenic mice exhibit cognitive findings before amyloid deposition is present (as shown by the NOR testing in this study) and learning behavior impairments tend to progress in parallel with amyloid plaque deposition (Howlett et al., 2004; Morgan, 2006). Transgenic mice also typically lack the tau pathology and synaptic loss characteristic of human AD (Ashe and Zahs, 2010). Yet despite the imperfect correlation between mouse models and human AD, the current study suggests that BCI-838 and exercise might be beneficial in the pre-symptomatic and/or MCI phases of AD. Interestingly, BCI-838 also reduces Aβ monomers and oligomers (Kim et al., 2014). Thus, BCI-838 could be acting through multiple mechanisms, some amyloid dependent and others not. Future studies in APP/PS1 mice at older ages (e.g., 5 or 6 months) when levels of oligomeric Aβ and amyloid deposition are increasing rapidly will be of interest. More extended treatment regimens (e.g., 2–3 months) beginning at 3 months of age (or earlier) could provide support for the notion that BCI-838 might be useful for some form of prophylaxis in humans.

Alone or in combination, BCI-838 administration and PE enhanced hippocampal neurogenesis. This conclusion was supported both by BrdU injections given 24 h before sacrifice (which provided a measure of neural progenitor proliferation) and by counts of DCX-labeled cells, which provided a measure of the steady-state population of young neurons. Using either measure, BrdU- and DCX-labeled cells were increased following drug treatment or the combination of exercise and drug treatment when compared to APP/PS1 mice treated with vehicle. However, comparing BCI-838 alone to the combination group did not lead to any obvious additional benefit of adding BCI-838 to PE. We interpret this as suggesting that BCI-838 acts to mimic aspects of exercise's effects rather than to act synergistically to enhance it. The fact that no DEGs were observed when comparing the combination group with BCI-838 alone further supports the notion that the BCI-838 effect is to mimic rather than synergistically enhance PE.

Of note, in our studies, neurogenesis was not impaired in APP/PS1 mice treated with vehicle when compared to WT mice. Prior studies in AD transgenic mice have produced inconsistent results concerning the effect of familial AD mutations on AHN (Jin et al., 2004; Wen et al., 2004; Kuhn et al., 2007; Taniuchi et al., 2007; Verret et al., 2007; Niidome et al., 2008; Yu et al., 2009; Chuang, 2010; Demars et al., 2010; Elder et al., 2010; Krezymon et al., 2013; Unger et al., 2016), including in the APP/PS1 mice studied here (Taniuchi et al., 2007; Verret et al., 2007; Niidome et al., 2008; Yu et al., 2009; Chuang, 2010; Unger et al., 2016). The absence of impaired AHN in APP/PS1 mice in this study, compared to WT mice suggests that the effect of BCI-838 and exercise on the neurogenesis may be an enhancement of baseline function rather than restoration of disease-related deficiency. This point may be important in determining whether PE is most effective administered as prophylaxis vs. retardation of symptom progression.

Most studies suggest that voluntary running alone increases adult neural progenitor proliferation, while other manipulations such as environmental enrichment favor survival of adult-born dentate granule neurons and increased integration of immature neurons into neuronal circuits (Van Praag et al., 2005; Ramirez-Amaya et al., 2006; Toda et al., 2019). Learning itself also promotes functional integration of new neurons into behaviorally relevant networks, in particular recruiting higher proportions of younger rather than older born cells into hippocampal assemblies (Ramirez-Amaya et al., 2006). Additional studies will be required to determine the fate of newly generated cells and whether BCI-838 and/or PE stimulate the production of new neurons that become integrated into functional neuronal networks as well as whether enhancement of neurogenesis is required for the effects of BCI-838 with or without PE.

Why an mGluR2/3 receptor antagonist should mimic some effects of PE is unclear. PE exerts a variety of beneficial effects on neuroplasticity, spatial learning, and memory (Caselli and Reiman, 2013). Neurogenesis in adult brain is likely regulated through a combination of factors (Obernier and Alvarez-Buylla, 2019), and PE also acts on multiple pathways (Buchman et al., 2019). Mechanistically, many studies have emphasized PE-related effects on trophic factor production, especially BDNF, insulin-like growth factor-1 (IGF-1), and vascular endothelial growth factor (VEGF) (Cassilhas et al., 2016). A recent study in AD transgenic 5xFAD mice further demonstrated that exercise-induced AHN improved cognition along with increased levels of BDNF (Choi et al., 2018).

More recent work has emphasized how exercise affects secreted factors in the periphery (Cooper et al., 2018). Myokines secreted by muscle during exercise improve learning and memory by regulating hippocampal function (Lourenco et al., 2019; Pedersen, 2019). Cathepsin B, a PE-related myokine, crosses the blood-brain barrier and enhances BDNF production (Lourenco et al., 2019). Furthermore, irisin, the cleaved and circulating form of the exercise-induced fibronectin type III domain-containing membrane protein (FNDC5), stimulates BDNF production (Rabiee et al., 2020; Islam et al., 2021); its genetic deletion was shown to impair cognitive function in exercise, aging and AD; and its peripheral application was observed to be sufficient to rescue the learning behavior decline in mouse models of AD (Islam et al., 2021). We did not detect FNDC5 as a DEG in the DG of the APP/PSEN1 either following BCI-838 administration or after PE vs. sedentary, which may be due to the relatively young age of the APP/PS1 mice used and the absence of neuronal and synaptic loss at this age in this model. The length of voluntary PE may also have been a factor.

Indeed, one of the most important recent clues to the molecular basis for PE may be the discovery of irisin by Lourenco et al. (2019). This was recently confirmed and extended by Islam et al. (2021). Moreover, while our paper was under review, Kam et al. (2022), reported that irisin can ameliorate synuclein pathology in mouse models of Parkinson's disease. Integrins are receptors for irisin in bone and fat cells (Kim et al., 2018). Single-cell RNA-seq data from the murine DG found ITGAV expressed in neuronal and non-neuronal cells and ITGB5 expressed in astrocytes and microglia, raising the possibility that irisin might mediate glia activation through integrin receptor complexes (Islam et al., 2021). When we scrutinized our data for possible evidence of transcriptomic changes among integrin family members, we observed upregulation of ITGB8, an ECM receptor that regulates neurogenesis and neurovascular homeostasis in the adult brain (Mobley et al., 2009), following BCI-838 administration to APP/PS1 mice, at an FDR of 5%, as well as ITGB6 and ITGB7 at an FDR of 1%. Future studies will be needed to determine whether the effects of BCI-838 may be through irisin-related signaling (Rabiee et al., 2020).

mGluR2/3 receptors function primarily as presynaptic autoreceptors that, when stimulated, inhibit glutamate release (Koike et al., 2011; Chaki, 2019). The antidepressant effects of agents acting on the mGlu2/3 receptor have been widely studied in animal models. mGlu2/3 receptor blockade appears to act at least in part through mTOR signaling, which may contribute to the sustained antidepressant-like effects of mGlu2/3 receptor antagonists (Chaki and Fukumoto, 2018). However, the full synaptic and neural mechanisms of their action (including how mGluR2/3 antagonists stimulate AHN) remain to be clarified (Koike et al., 2011; Kim et al., 2019).

In this study, PE did not significantly influence AHN. The study did not include a group of WT mice treated with PE alone. Therefore, there was no internal control for the effect of PE on AHN in WT mice. The effect of PE on AHN in transgenic AD models has not been consistent. In 5x FAD mice, 4 months of voluntary PE was found to increase AHN (Choi et al., 2018). Two studies found increased AHN in APP/PS1 mice (Falkenhain et al., 2020; Yu et al., 2021). However, another study found that PE did not alter the number of BrdU positive cells in the dentate gyrus of APP/PS1 mice (Zhang et al., 2022). In our own studies, we saw a trend toward PE increasing anti-doublecortin (anti-DCX) labeled cells in the DG of APP/PS1 mice treated with PE alone (Figure 4), but this trend only reached statistical significance if no correction for multiple comparisons was made. By contrast, BCI-838 or BCI-838 + PE clearly increased the number of DCX and BrdU labeled cells (Figure 4). Differences between our studies and other studies may reflect differences in the model(s) used (e.g., 5x FAD vs. APP/PS1) as well as differences in ages of the animals studied, the PE interval, and whether PE was voluntary or forced.

Work in transgenic 5xFAD mice (Choi et al., 2018) revealed that chronic voluntary PE in running wheels for 4 months, beginning at 2 months of age, improved learning behavior in 5xFAD mice; our study revealed improvement in 1 month of PE. PE-mediated enhancement of neurogenesis in hippocampus is suggested to be mediated through BDNF signaling (Cooper et al., 2018). In our study, while a short treatment with BCI-838 or PE was associated with increased neurogenesis in the DG, the trend toward increased levels of BDNF did not reach statistical significance. However, APP/PS1 mice treated with BCI-838 and PE displayed indistinguishable levels of BDNF when compared with PE-treated mice, even though they spent less time on running wheels. This effect shows the potential of BCI-838 to mimic the effects of PE on BDNF expression in the DG. Future studies with larger sample sizes will be needed to better establish the effects of BCI-838 on BDNF levels in relationship to PE.

While attractive as a contributing factor, modulation of hippocampal levels of BDNF is unlikely to provide a complete explanation for the effects we observed. Unlike treatment with P7C3 studied by Choi et al. (2018), BCI-838 stimulated AHN and improved recognition memory without addition of PE. Thus BCI-838 may provide a pharmacological mimic of some aspects of PE, in its effects on behavior.

Our findings further demonstrate that administration of BCI-838 alone induced transcriptomic changes that resulted in up-regulation of BDNF and PIK3C2A of mTOR signaling, down-regulation of IL11RA1 interleukin receptor of AKT signaling, and enriched SP1 and further relevant pathways, implicating neurogenesis and effects of PE. These findings are especially notable as the mTOR pathway is involved in many aspects of neurogenesis as a regulator of cellular energy metabolism, a nutrient sensor and growth factor inducer via insulin, IGF-1 and BDNF (LiCausi and Hartman, 2018; Querfurth and Lee, 2021). The mTORC1 protein complex controls protein synthesis by phosphorylating downstream targets essential for mRNA translation, 4E-BP1 (eIF-4E binding protein) and ribosome biogenesis (Swiech et al., 2008). Neuronal growth factors known to support learning and memory such as BDNF and EGF do so through mTOR activation. Furthermore, in relation to the AD brain being an insulin-resistant organ, mTORC1 has been shown to be involved with down-regulating insulin/Akt signaling through an inactivating phosphorylation of IRS-1 (Tzatsos and Kandror, 2006).

Mechanistically, BCI-838 is considered an orally active ketamine-mimetic, and recent evidence indicates that ketamine modulates mTOR activity and the actions of the physiological effectors of elongation inhibitory factor 4E (eIF4E) and its protein partners, eIF4E binding proteins (eIF4E-BPs). This is also consistent with our findings of dysregulation of a related eukaryotic translation initiation factor, EIF5A. mTOR is known to modulate autophagy (Dossou and Basu, 2019). Given the behavioral benefits of BCI-838 in multiple types of proteopathies (Perez-Garcia et al., 2023) its apparent similarity to ketamine regarding mTOR modulation, and the role for mTOR in autophagy, we propose that, like ketamine, BCI-838 exerts its actions through protein translation and autophagy.

A drug repurposing analysis identified several known compounds transcriptomically similar to BCI-838. Of these compounds some have been suggested as having a potential role in AD pathophysiology or therapy. In particular, meptazinol derivatives are dual inhibitors of cholinesterases and amyloid-beta aggregation with multiple studies showing effectiveness of these derivatives in APP/PS1 mice (Liu et al., 2013; Shao et al., 2014; Shi et al., 2018; Wang et al., 2019). Several studies have identified a puromycin-sensitive aminopeptidase as a potential inhibitor of tau (Karsten et al., 2006; Sengupta et al., 2006; Hui, 2007; Kudo et al., 2011) and Aβ related neurodegeneration (Kruppa et al., 2013). Why puromycin which inhibits this aminopeptidase (Constam et al., 1995) should have transcriptional effects similar to BCI-838 is unclear. Gramine derivatives have been suggested to potentially exert neuroprotective effects in neurodegenerative disorders including AD through their ability to ameliorate calcium overload and modify serine/threonine proteases (Lajarin-Cuesta et al., 2016; Gonzalez et al., 2018). MG-132 is a proteosome inhibitor commonly used to experimentally induce proteinopathy related neurodegeneration (Posimo et al., 2015; Heinemann et al., 2016) although in one study MG-132 restored impaired activity-dependent synaptic plasticity as well as associative long-term memory in APP/PS1 mice, an effect possibly mediated through stimulation of the mTOR pathway (Krishna et al., 2020). Future studies will be needed to understand the complex transcriptional effects of BCI-838 and these related compounds.

Previous studies explored the effects of PE or mGluR2/3 modulation alone on AD-related pathology (Kim et al., 2014; Tapia-Rojas et al., 2016). However, none explored the effect of the combination of mGluR2/3 modulation and PE in the early stages of AD development in a mouse model. Here we show that BCI-838 improved behavior in APP/PS1 mice and stimulated AHN. Combined with our previous studies in another AD mouse model (Kim et al., 2014), a rat model of blast-related traumatic brain injury (Perez-Garcia et al., 2018) and the PS19 MAPTP301S mouse model of tauopathy (Perez-Garcia et al., 2023), four preclinical studies now support the effectiveness of BCI-838 for relief of a range of models of neurocognitive, neurobehavioral, and neurodegenerative traits. The ability of BCI-838 to recapitulate many of the effects of PE suggests that this and/or other mGluR2/3 antagonists may represent safe and novel pharmacological mimics of PE. If this notion regarding pharmacological mimicry of PE proves to be true, it will be worth evaluating whether these drugs can prevent, delay, and/or treat neurodegenerative and/or post-traumatic disorders of cognition, anxiety, and/or behavior.

Several study limitations should be mentioned. The experimental design did not include non-transgenic wild type mice treated with BCI-838. The rationale for this exclusion was to limit animal usage to the minimum needed to achieve the experimental goal of determining the effects of BCI-838 on behavior in APP/PS1 mice. In the design utilized (Figure 1), we reasoned that comparison of the single group of vehicle treated WT mice (group 5) to vehicle treated APP/PS1 mice (group 1) served as a positive control for appearance of the transgene-related behavioral phenotype while comparison of BCI-838 +/– PE treated APP/PS1 mice (groups 2–4) to vehicle treated APP/PS1 mice (group 1) measured effectiveness of BCI-838 treatment.

The BCI-838 effect is unlikely to be transgene-specific in that as noted above BCI-838 has been shown to rescue behavioral deficits in a transgenic model using a different AD transgene (Kim et al., 2014). BCI-838 also rescued behavioral deficits in wild type rats subjected to blast exposure (Perez-Garcia et al., 2018) and recently proved effective in reversing impaired recognition memory in the PS19 MAPTP301S mouse model of tauopathy (Perez-Garcia et al., 2023). In addition, as a class, mGluR2/3 receptor antagonists show anti-depressant like action in a variety of stress induced models using wild type mice or rats (Chaki, 2019). We note that in the previous study utilizing Dutch APP (*APP*^*E*693*Q*^) transgenic mice, while BCI-838 improved learning in BCI-838 treated Dutch APP mice, the drug had no effect on NOR or cued fear learning in non-transgenic wild type mice (Kim et al., 2014).

In addition, we do not know how behavior may affect drug, exercise and transgene interactions on RNA transcriptomics. In this study, a conscious decision was made not to perform transcriptional analysis on mice that had undergone behavioral testing. This decision was made to focus attention on the static state changes induced by the drug, exercise, and drug/exercise interactions alone without the added complication of behavioral interactions. The transcriptional studies provide a correlation with behavior in that they represent the state of the animals before they underwent behavioral testing. However, in humans, there are always effects of behavioral interactions. It therefore will be of interest to perform additional transcriptomics studies after selected behavioral testing. To understand BCI-838's potentially complex effects, the study of additional BCI-838 treated WT mice will be essential. The choice of these studies will be facilitated by results of the present work.

The conclusions of this study are also limited by the restricted number of behavioral tests that were performed. In particular, anxiety and depression are now understood to be core clinical features of AD, and both could affect performance in cognitive tasks. Future studies including an expanded range of behavioral tests will be needed to more fully understand BCI-838's effects. A final limitation of the current studies is that we only included male mice, which reduces the generalizability of the findings. There indeed may be sex-specific differences in response to BCI-838, and there are sex differences in amyloid deposition in APP/PS1 mice (Wang et al., 2003). Future studies in female mice will be important.

Lifestyle modification is being widely studied as a disease-modifying intervention for AD. PE was a major component of the FINGER trial (Ngandu et al., 2015; Wimo et al., 2022). Thus understanding the action of compounds like BCI-838, which apparently comprise a combination of PE-related and PE-independent effects, offers new opportunities to better illuminate these mechanisms, perhaps extend them and inform the development of new therapeutic strategies for this devastating disease.

While we do not mean to imply that BCI-838 could or should replace PE in otherwise healthy individuals, there are many individuals with chronic medical or physical conditions that limit their full participation in regular PE programs. For such individuals, a drug like BCI-838 might be a reasonable way to supplement an inability to fully participate in a formal PE program. In humans, the application of PE may well need to be individualized in relationship to diet, lifestyle, genetic background, and education, all goals of modern personalized medicine, making it perhaps even more important to understand the effects of PE alone in an animal model where these effects can be isolated.

The pharmacokinetics, safety and tolerability of BCI-838 have been evaluated through phase 1 human studies, which found BCI-838 well tolerated in healthy subjects without serious adverse effects (Clinicaltrials.gov1, 2012; Clinicaltrials.gov2, 2012). Thus, data from both experimental animals and humans suggest that BCI-838 is safe and ready to be advanced into human clinical trials.

## Conclusion

PE has beneficial neuroprotective and pro-cognitive effects including stimulating AHN. PE has been widely studied as an intervention that may delay the development of AD. Here we show that BCI-838 improved recognition memory and enhanced AHN in an APP/PS1 mouse AD model. Transcriptional analysis of the hippocampal dentate gyrus following BCI-838 treatment showed up-regulation of brain BDNF, PIK3C2A of the PI3K-mTOR pathway and down-regulation of EIF5A a modulator mTOR activity. BCI-838 is thus a safe orally active compound capable of mimicking some of the beneficial effects of PE.

## Data availability statement

The original contributions presented in the study are publicly available. This data can be found here: https://www.ncbi.nlm.nih.gov/, GSE203554.

## Ethics statement

The animal study was reviewed and approved by Institutional Animal Care and Use Committee (IACUC) of the James J. Peters VA Medical Center.

## Author contributions

CB and FHG identified the key pro-neurogenic action of BCI-838 and supplied the drug for these studies. ME, SG, GPG, JD, and GE designed the study. GPG, J-VH-M, GP, and AO-P performed the experiments. GPG, BR, MB, and GE analyzed data. GPG, MB, SG, ME, JD, J-VH-M, BG, BR, MS, and GE wrote the paper. All authors have read and approved the final manuscript.
