# Supplementary material for: Beneficial effects of physical exercise and an orally active mGluR2/3 antagonist pro-drug on neurogenesis and behavior in an Alzheimer's amyloidosis model
Source: Front Dement. 2023 Sep 6;2:1198006. doi: 10.3389/frdem.2023.1198006 (PMC11285632; doi:10.3389/frdem.2023.1198006)
Supplement: Supplementary file 2 [file Table_2.PDF]

Supplementary Table 2. Gene set enrichment analysis results

**Names of group comparisons in online data set**	**Names used in the manuscript**
BorControl vs WT	APP/PS1 untreated vs Wild type
BorBCI vs BorControl	APP/PS1 treated with BCI-838 vs APP/PS1 untreated
BorPE vs BorControl	APP/PS1 treated with PE vs APP/PS1 untreated
BorCombination vs BorControl	APP/PS1 treated with BCI-838 and PE vs APP/PS1 untreated
BorCombination vs BorBCI	APP/PS1 treated with BCI-838 and PE vs APP/PS1 treated with BCI-838
BorCombination vs BorPE	APP/PS1 treated with BCI-838 and PE vs APP/PS1 treated with PE
