# Supplementary material for: Beneficial effects of physical exercise and an orally active mGluR2/3 antagonist pro-drug on neurogenesis and behavior in an Alzheimer's amyloidosis model
Source: Front Dement. 2023 Sep 6;2:1198006. doi: 10.3389/frdem.2023.1198006 (PMC11285632; doi:10.3389/frdem.2023.1198006)
Supplement: Supplementary file 3 [file Table_3.PDF]

Supplementary Table 3.

Drug repurposing results

Query_drugs
Targets_DB41
Enzymes_DB41
Transporters_DB41
Carriers_DB41
Targets_SEA
Fragments
SideFX_SIDER2
SideFX_Offsides
Drug_class_ATC3
Drug_class_ATC4
Disease_Indications
