# Supplementary material for: Beneficial effects of physical exercise and an orally active mGluR2/3 antagonist pro-drug on neurogenesis and behavior in an Alzheimer's amyloidosis model
Source: Front Dement. 2023 Sep 6;2:1198006. doi: 10.3389/frdem.2023.1198006 (PMC11285632; doi:10.3389/frdem.2023.1198006)

Supplemental Figure 1. Variance partitioning results. **a** Violin plot of all genes. **b** Variation plot of top 50 genes.

a.

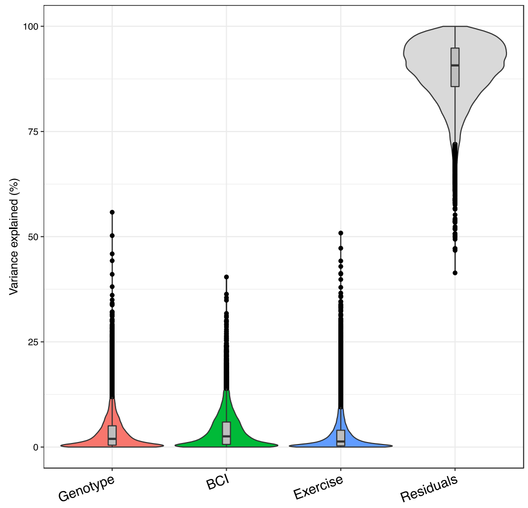

b.

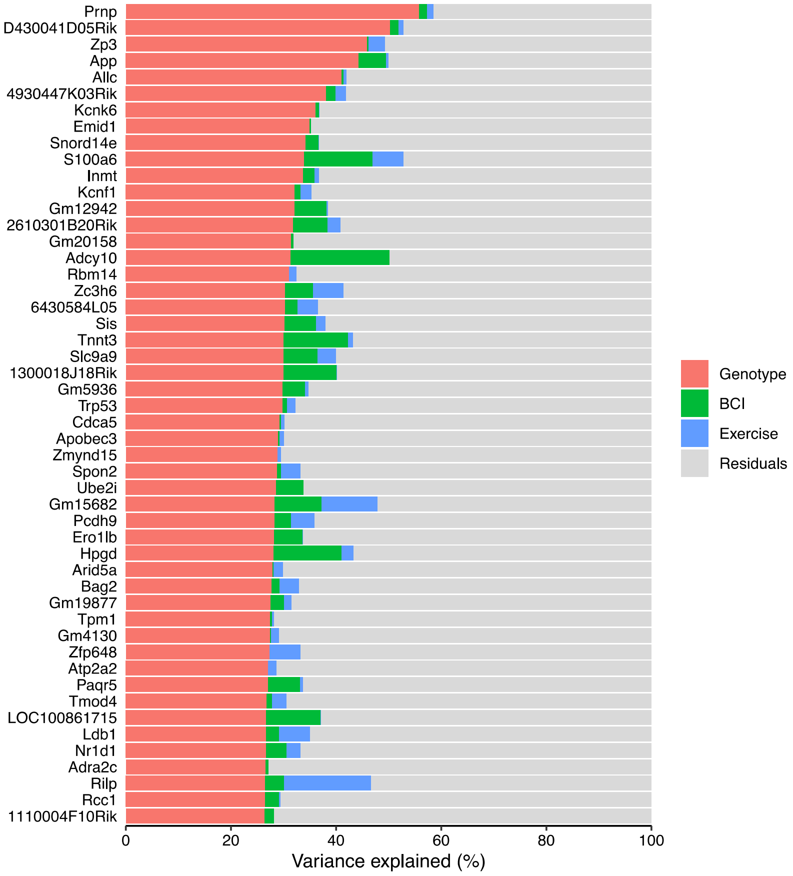

Supplement: Supplementary file 4 [file Image_1.PDF]
